# Genetic variations in apoptosis pathway and the risk of ovarian cancer

**DOI:** 10.18632/oncotarget.10772

**Published:** 2016-07-22

**Authors:** Hui Xie, Wade Tao, Xifeng Wu, Jian Gu

**Affiliations:** ^1^ Department of Breast Surgery, The First Affiliated Hospital of Nanjing Medical University, Nanjing, China; ^2^ Department of Epidemiology, The University of Texas MD Anderson Cancer Center, Houston, Texas, USA

**Keywords:** single nucleotide polymorphism, apoptosis pathway, ovarian cancer, risk

## Abstract

**Background:**

Apoptosis is a highly conserved form of cell death and aberrant regulation of apoptotic cell death mechanisms leads to variety of major human diseases, especially tumor formation. Genetic variations in apoptosis genes may increase susceptibility to ovarian cancer.

**Results:**

In individual SNP analysis, 12 SNPs in 5 apoptosis pathway genes were significantly associated with ovarian cancer risk after adjustment for multiple comparisons at q-value <0.05. The most significant SNP was rs11152377 in the *Bcl-2* gene. The homozygous variant TT genotype was associated with a significantly decreased risk of ovarian cancer (odds ratio [OR] =0.53; 95% confidence interval [CI], 0.37-0.77, P<0.001). Cumulative effect analysis showed joint effects of increased risk of ovarian cancer with increasing number of unfavorable genotypes in patients. Classification and regression tree (CART) analysis further revealed high-order gene-gene interactions and categorized the study subjects into low-, medium-, and high-risk groups. Compared with the low-risk group, medium-risk group and high-risk group conferred 1.76-fold (95% CI: 1.06–2.90) and 3.64-fold (95% CI: 2.37–5.59) increased risk of ovarian cancer (P for trend <0.001)

**Materials and Methods:**

In a case-control study of 417 ovarian cancer patients and 417 matched controls, we evaluated the associations of 587 single nucleotide polymorphisms (SNPs) from 65 genes of the apoptosis pathway with the risk of ovarian cancer.

**Conclusions:**

Our results suggest that genetic variations in apoptosis pathway genes modulate the risk of ovarian cancer individually and jointly.

## INTRODUCTION

Ovarian cancer is the fifth most frequently leading cause of cancer deaths and remains the most lethal of all gynecologic malignancies in women in the United States. Approximately, 22,280 new cases and 14,240 deaths were estimated in 2016 [1]. Most women with ovarian cancer are diagnosed with advanced disease (stage III or IV) and have only 10–30% survival rates [2, 3]. The main risk factors for ovarian cancer are all hormone-related, including parity, oral contraceptive use, and age of menarche and menopause [4]. Smoking and alcohol drinking are not associated with epithelial ovarian cancer, but obesity, physical activity and dietary intake may be associated with ovarian cancer [4]. Family history is an important risk factor of ovarian cancer pointing to genetic susceptibility of this disease [5]. It is well known that BRCA1 and BRCA2 mutations are the most frequent hereditary risk factor [6]. DNA mismatch repair genes such as MSH2 or MLH1 also play important roles in inherited ovarian cancer [7]. Recent genome-wide association studies (GWAS) have identify nearly 20 common genetic susceptibility loci for ovarian cancer [8, 9]. In the ear of GWAS, pathway-based approach is a complementary method to identify novel genetic susceptibility loci for diseases including cancer. By focusing on specific biological pathways that are closely related to disease pathogenesis, pathway-based approaches require smaller sample sizes, offer higher genomic coverage, and more reliably find gene-gene interactions and gene networks than GWAS. Many recent studies have demonstrated the value of the pathway-based approach in identifying novel genetic susceptibility SNPs for cancer risk [10–16].

Multicellular organisms maintain homoeostasis by the controlled elimination of cells that are no longer needed or are damaged by a cell suicide pathway known as apoptosis [17]. Apoptosis is a highly conserved form of cell death and aberrant regulation of apoptotic cell death mechanisms leads to a variety of human diseases, especially cancer [18]. Previous molecular epidemiologic studies have shown that SNPs in apoptosis pathway are associated with the risks of different cancers [19–22]. However, the association of SNPs in the apoptosis pathway with the risk of ovarian cancer has not been systematically studied yet.

In this case-control study, we applied a pathway-based approach to evaluate a large number of single nucleotide polymorphisms (SNPs) in major apoptosis genes as genetic susceptibility loci for ovarian cancer and also explored high-order gene-gene interactions in the apoptosis pathway in affecting ovarian cancer susceptibility.

## RESULTS

### Characteristics of the study population

A total of 417 patients diagnosed with ovarian cancer cases and 417 controls were included in this study. Control subjects were matched to the cases by age (±5 year) and ethnicity. The mean ages (± SDs) of cases and controls were 60.73 ± 10.36 and 60.30 ± 10.71, respectively. No significant differences were observed between cases and controls for age (P = 0.554) and ethnicity (P = 0.269). The major of ethnicity were Caucasians (81.3% in cases vs. 83.7% in controls) (Table 1).

**Table 1:** Basic characteristics of cases and controls

Category	Subcategory	Cases, N (%)	Controls, N (%)	P
Age, Mean (SD^*^)		60.7 (10.4)	60.3 (10.7)	0.554
**Ethnicity**	White	339 (81.3)	349 (83.7)	0.269
	Hispanic	48 (11.5)	49 (11.8)	
	Black	20 (4.8)	15 (3.6)	
	Other	10 (2.4)	4 (0.9)	
**Smoking status**	Never	279 (68.6)	285 (68.4)	0.957
	Former	93 (22.8)	98 (23.5)	
	Current	35 (8.6)	34 (8.1)	

* SD: standard deviation.

### Association between individual SNP variant genotype and risk of ovarian cancer

The analyses included 587 SNPs within 65 genes of the apoptosis pathway. We discarded 5 SNPs that showed significant departure from Hardy–Weinberg equilibrium (P < 0.01). A total of 46 SNPs showed significant association with ovarian cancer risk at P<0.05. Among 46 SNPs with significant main effects, 12 SNPs in 5 genes remained significant after adjustment for multiple comparisons at q-value <0.05. SNP rs11152377 in BCL-2 gene showed the most significant effect on ovarian cancer risk with a recessive model (Table 2). The homozygous variant TT genotype was associated with a 0.53 fold decreased risk of ovarian cancer (odds ratio [OR] =0.53; 95% confidence interval [CI], 0.37-0.77, P<0.001). Among these 12 SNPs, 11 SNPs exhibited considerably reduced risk. Only rs2889 located in TNFRSF10B exhibited considerably increased risk in a dominant model. To internally validate the associations, we performed bootstrap resampling for 100 times and listed the number of times for each SNP that bootstrap-generated P value was <0.05, p<0.01, p<0.001. The overall odds ratios and 95% CIs generated by bootstrapping were consistent with our initial results. All these 12 SNPs exhibited highly consistent results with P<0.05 at 100 times in 100 bootstrap samples. This indicates that the results for these SNPs are unlikely to be due to chance alone. All of these SNPs, with the exception of rs1801018 on BCL-2 that is a coding non-synonymous SNP, are either intronic or intergenic, because most of the SNPs were tagging SNPs.

**Table 2:** Significant individual SNPs associated with ovarian cancer risk

Gene SNP	Genotype	Cases, N(%)	Controls, N(%)	Adjusted OR (95% CI)^*^	P value	Best fitting Model^#^	Q value	Bootstrap		
								P <0.05	P <0.01	P <0.001
**BCL-2**	CC+CT	364 (87.29)	326 (78.18)	1 (reference)						
**rs11152377**	TT	53 (12.71)	91 (21.82)	0.53 (0.37-0.77)	**<0.001**	Recessive	**0.037**	100	99	49
**BIK**	AA	150 (35.97)	119 (28.74)	1 (reference)						
**rs135014**	AG	199 (47.72)	189 (45.65)							
	GG	68 (16.31)	106 (25.60)							
	p for trend			0.73 (0.60-0.89)	**0.001**	Additive	**0.037**	100	99	32
**TP53BP1**	AA	308 (74.04)	268 (64.27)	1 (reference)						
**rs16957730**	AG+GG	108 (25.96)	149 (35.73)	0.63 (0.47-0.85)	**0.002**	Dominant	**0.037**	100	95	28
**TNFRSF10B**	CC	167 (40.14)	209 (50.12)	1 (reference)						
**rs2889**	CT+TT	249 (59.86)	208 (49.88)	1.53 (1.16-2.02)	**0.002**	Dominant	**0.037**	100	97	25
**TP53BP1**	CC	378 (90.65)	348 (83.45)	1 (reference)						
**rs17782975**	CT+TT	39 (9.35)	69 (16.55)	0.53 (0.35-0.81)	**0.003**	Dominant	**0.037**	100	92	16
**BCL-2**	AA+AG	353 (84.65)	318 (76.26)	1 (reference)						
**rs1801018**	GG	64 (15.35)	99 (23.74)	0.60 (0.42-0.85)	**0.004**	Recessive	**0.037**	100	81	6
**BIRC5**	CC+CG	394 (94.49)	373 (89.67)	1 (reference)						
**rs744120**	GG	23 (5.52)	43 (10.34)	0.45 (0.26-0.78)	**0.004**	Recessive	**0.037**	100	74	3
**BCL-2**	AA	348 (83.65)	322 (77.40)	1 (reference)						
**rs1016860**	AG	66 (15.87)	84 (20.19)							
	GG	2 (0.48)	10 (2.40)							
	p for trend			0.63 (0.46-0.87)	**0.005**	Additive	**0.037**	100	91	11
**BCL-2**	AA+AG	340 (81.53)	306 (73.38)	1 (reference)						
**rs4941183**	GG	77 (18.47)	111 (26.62)	0.63 (0.45-0.88)	**0.007**	Recessive	**0.045**	100	75	6
**BIK**	CC	337 (80.82)	302 (72.42)	1 (reference)						
**rs4988360**	CT+TT	80 (19.19)	115 (27.58)	0.64 (0.46-0.89)	**0.007**	Dominant	**0.045**	100	80	1
**BIK**	GG+GT	339 (81.29)	305 (73.54)	1 (reference)						
**rs5759167**	TT	78 (18.71)	112 (26.86)	0.64 (0.46-0.89)	**0.008**	Recessive	**0.045**	100	57	2
**TNFRSF10B**	AA+AG	363 (90.75)	338 (85.57)	1 (reference)						
**rs1001793**	GG	37 (9.25)	57 (14.43)	0.54 (0.35-0.86)	**0.008**	Recessive	**0.046**	100	56	3

* Adjusted by age, ethnicity, and smoking status.

# Internal validation of the results choosing from the best genetic model using bootstrap for 100 times.

### Cumulative effect of unfavorable genotypes of apoptosis genes

To further assess the cumulative effect of multiple SNPs in apoptosis genes associated with ovarian cancer risk, we conducted the unfavorable genotype analysis by distributing individuals into separate risk groups and analyzed the resulting association with ovarian cancer risk (Table 3). We divided the subjects into four risk subgroups according to the quartile of overall subject investigated. We found a statistically significant trend toward an increasing gene-dosage effect for ovarian cancer risk associated with an increasing number of unfavorable genotypes. That is, compared with reference group (group 1 with 3-7 unfavorable genotypes), group 2 (with 8-9 unfavorable genotypes), group 3 (with 10 unfavorable genotypes), and group 4 (with 11-12 unfavorable genotypes) had a progressively increased ovarian cancer risk with ORs of 2.62 (95% CI, 1.52-4.51), 4.25 (95% CI, 2.43-7.45), and 7.42 (95% CI, 4.23-13.04), respectively (p for trend <0.001).

**Table 3:** Joint effects of unfavorable genotypes in apoptosis pathway genes on the risk of ovarian cancer

Number of unfavorable genotypes	Cases, N (%)	Controls, N (%)	Adjusted OR^*^	P value
**3~7**	21 (22.11)	74 (77.89)	1 (reference)	
**8~9**	115 (42.44)	156 (57.56)	2.62 (1.52-4.51)	**<0.001**
**10**	110 (54.73)	91 (45.27)	4.25 (2.43-7.45)	**<0.001**
**11~12**	151 (67.11)	74 (32.89)	7.42 (4.23-13.04)	**<0.001**
**p for trend**				**<0.001**

* Adjusted by age, ethnicity, and smoking status.

### CART analysis

**Figure 1:**
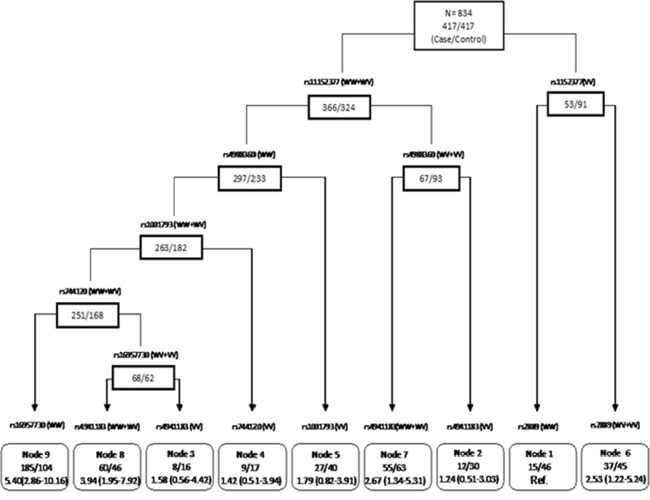
CART analysis of genetic polymorphisms in the apoptosis pathway and risk of ovarian cancer For each SNP, “WW” represents wild-type, “WV” represents heterozygous genotype, and “VV” represents homozygous variant genotype.

We then applied the CART analysis by using a binary recursive-partitioning method, which identifies subgroups of high-risk subjects and detects higher-order gene–gene interactions among a large number of variables. By examining genotypes of significant apoptosis SNPs (P< 0.05 in the best-fitting model) identified from the individual SNP analysis as attributes for tree construction, a resulting tree with nine terminal nodes was generated (Figure 1). The initial split was rs11152377 of BCL-2, the most significant SNP out of those evaluated for ovarian cancer risk. Terminal node 1 was defined as individuals carrying the homozygous variant genotypes (VV) for rs11152377 of BCL-2, following by the homozygous wildtype (WW) for rs2889 of TNFRSF10B, which had the lowest ovarian cancer risk as the reference node. Subjects with the highest ovarian cancer risk were those individuals in node 9 with genotypes of the homozygous wildtype or heterozygous genotype (WW/WV) for rs11152377 of BCL-2, following by WW for rs4988360 of BIK, WW/WV for rs1001793 of TNFRSF10B, WW/WV for rs744120 of BIRC5, and WW for rs16957730 of TP53BP1. Compared to node 1, the ORs of terminal node 2 to 8 ranged from 1.25 (95% CI 0.51–3.03) to 5.40 (95% CI 2.86-10.16) based on distinct SNP genotype combinations (Table 4).

**Table 4:** CART terminal nodes and ovarian cancer risk

Terminal node*	Cases, N (%)	Controls, N (%)	Adjusted OR^#^	P value
**Node 1**	15 (24.59)	46 (75.41)	1 (reference)	
**Node 2**	12 (28.57)	30 (71.43)	1.25 (0.51-3.03)	0.629
**Node 3**	8 (33.33)	16 (66.67)	1.58 (0.56-4.42)	0.388
**Node 4**	9 (34.62)	17 (65.38)	1.42 (0.51-3.94)	0.501
**Node 5**	27 (40.30)	40 (59.70)	1.79 (0.82-3.91)	**0.143**
**Node 6**	37 (45.12)	45 (54.88)	2.53 (1.22-5.24)	**0.012**
**Node 7**	55 (46.61)	63 (53.39)	2.67 (1.34-5.31)	**0.005**
**Node 8**	60 (56.60)	46 (43.40)	3.94 (1.95-7.92)	**<0.001**
**Node 9**	185 (64.01)	104 (35.99)	5.40 (2.86-10.16)	**<0.001**
p for trend				**<0.001**

## DISCUSSION

In this case–control study, we systematically assessed the association between a panel of genetic polymorphisms involved in the apoptosis pathway and risk of ovarian cancer. We screened 587 SNPs located in 65 apoptotic-pathway genes in a case-control study. We found that 46 SNPs showed significant associations with ovarian cancer risk in the discovery population and 12 SNPs remained significant after adjustment for multiple comparisons at q-value <0.05. The results from cumulative analysis further suggested that these genetic variants may influence ovarian cancer risk jointly, consistent with the polygenic etiology of ovarian cancer. Moreover, we also found potential high-order gene-gene interactions involved in the apoptosis pathway concerning ovarian cancer susceptibility that further defined high versus low risk subgroups in the study population.

Apoptosis is an essential cellular defense mechanism against cancer development [23, 24]. Apoptosis is triggered through two distinct molecular routes; the extrinsic or receptor-mediated pathway and intrinsic or mitochondrial pathways [25]. Both pathways involve the activation of a cascade of enzymes called caspases, a family of cysteine proteases that cleave substrates at aspartic acid residues [26]. The extrinsic and intrinsic pathways each have an independent group of “initiator” caspases, and the pathways converge on the same group of “effector” caspases to execute the cell death program. Intrinsic death stimuli, such as DNA damage, hypoxia, growth factor deprivation, or stress signals, can activate the intrinsic pathway resulting in the release of cytochrome c and the formation of the apoptosome complex consisting of cytochrome c, Apaf-1 and caspase-9 through members of the BCL-2 family [27–29]. Subsequently, caspase-9 cleaves and activates downstream enzymatic effector, such as caspases-3, −6, and −7, which then cleave the key regulatory and structural proteins to execute cell death [30]. The extrinsic apoptosis pathway is initiated by the activation of cell surface death receptors of the TNF receptor superfamily through binding of the extracellular ligands. Ligand binding to the extracellular domain of the death receptor results in receptor trimerization, with the subsequent recruit the adaptor protein Fas-associated death domain (FADD) and caspase-8 and/or caspase-10 to form a death-inducing signaling complex (DISC) in the intracellular death domain [31]. The activation of the caspase enzyme cascade leads to the unique morphological and biochemical cellular changes characteristic of apoptosis phenotypes such as membrane blebbing, nuclear condensation, DNA fragmentation, and ultimately phagocytosis by immune cells [32].

In this study, we found the SNP that most significantly associated with ovarian cancer risk is rs11152377 located in the BCL-2 gene. BCL-2 gene derives its name from B-cell lymphoma 2. It is the second member of a range of proteins initially described in chromosomal translocations involving chromosomes 14 and 18 in follicular lymphomas, placing BCL-2 under the control of the immunoglobulin heavy-chain promoter resulting in its deregulated high level of expression [33]. BCL-2 is one of the major pro-survival proteins that has an essential function in normal immunity and whose constitutive expression leads to the development of lymphomas. The BCL-2 gene promotes cellular survival rather than proliferation by inhibiting apoptosis and de-regulation of the gene induces overexpression of BCL-2 mRNA and the encoded protein, a phenomenon which has been observed in many solid cancers. Damage to the BCL-2 gene has been identified as a cause of a number of cancers, including melanoma, breast, prostate, leukemia, and lung cancer [34–38]. It is also a cause of resistance to cancer treatments [39, 40]. This SNP is an intronic SNP. Previous studies have shown that intonic SNPs may be functional, for example, intronic SNPs can alter RNA or DNA secondary structures [41, 42]. It is also likely that this SNP is a tagging SNP that tags other functional SNP(s). The molecular mechanisms underlying the association of this SNP with ovarian cancer risk warrants further study.

Our data showed that individual SNPs were only moderately associated with ovarian cancer risk, which is consistent with current literature of common SNPs and cancer risk [43]. However, we found a significant trend of increased risk with increasing numbers of unfavorable genotypes in the apoptosis pathway when the cumulative effects of genetic variations were assessed by using unfavorable genotype analysis. Ovarian cancer risk was higher in individuals with a higher number of adverse alleles than in individuals carrying a lower number of adverse alleles. These findings highlight the importance of using a multigenic approach to identify signatures of genetic variations as predictors of cancer risk.

We also performed CART analysis to define high-risk versus low-risk subgroups by exploring high-order gene-gene interactions among apoptosis pathway SNPs. Consistent with the main effect derived from the logistic regression analysis, the rs11152377 located in the BCL-2 gene was at the initial split, thereby suggesting that this variant functions as the primary determinant of ovarian cancer risk. The risk of ovarian cancer development in each node with distinct genotype profiles differed significantly, suggesting CART analysis has good discriminative ability. However, since CART analysis is a post-hoc data-mining tool and the number of subjects in the terminal nodes was small, these results should be interpreted with caution.

The major strength of this study is the large scale tagging SNP-based query of apoptosis pathway SNPs in a relatively large case control study. The cases and controls were matched on age, gender, and ethnicity. Nevertheless, our study has some limitations. The main limitation of this study is that due to the exploratory nature of this study, we did not perform more stringent multiple testing adjustment and we did not have an external validation. Further external validations in independent studies are warranted to confirm the results of the identified associations between apoptosis genes and ovarian cancer risk.

In conclusion, our study is the first study to apply a pathway-based approach to evaluate germline genetic variations in the apoptosis pathway and their associations with ovarian cancer risk. We support that common sequence variants of the apoptosis pathway genes may predispose individuals to increased risks of ovarian cancer. Future studies are needed to confirm out results and determine how these SNPs affect gene function and alter ovarian cancer susceptibility.

## MATERIALS AND METHODS

### Study subjects

Ovarian cancer cases were accrued from the University of Texas MD Anderson Cancer Center from August 1991 to January 2009. All patients were newly diagnosed and histologically confirmed ovarian cancer with no prior chemotherapy or radiotherapy treatment. There was no restriction on age, ethnicity, or disease stage in case recruitment. Control subjects were recruited in parallel with the cases from healthy individuals with no prior history of any type of cancer, except non-melanoma skin cancer, at Kelsey Seybold Clinic, the largest private multispecialty physician group in the Houston metropolitan area. Control subjects were frequency matched to the cases based on age (±5 years) and ethnicity. The response rate for cases was 90% and for controls 71%.

### Epidemiological and clinical data collection

Epidemiological data were collected based on a standardized questionnaire by trained MD Anderson Cancer Center staff. Data are collected on demographic characteristics (age, ethnicity, etc.), height, weight, body mass index, family history of cancer, medical history, working history, smoking status, and alcohol consumption. Ethnicity information was self-reported. After completion of the interview, a 40 ml blood sample was collected into heparinized tubes for immediate lymphocyte isolation and DNA extraction. Written informed consent was obtained from all patients before interview. The study was approved by the University of Texas MD Anderson Cancer Center and Kelsey Seybold institutional review boards. Written consent forms were obtained from patients before the interview.

### SNP selection and genotyping

We combined literature exploration and database of Gene Ontology (http://www.geneontology.org/) to select candidate genes in the apoptosis pathway. We also identified potentially functional SNPs, which are located in the functional regions of the genes, including coding (synonymous SNPs and nonsynonymous SNPs) and regulatory (promoter, splicing site, 5′-UTR, and 3′-UTR) regions. Tagging SNPs were then selected using the IDSelect program (http://droog.gs.washington.edu/ldSelect.html) to separate all of the selected SNPs into bins based on the linkage disequilibrium. Selected tagging SNPs have an r^2^ threshold of 0.8 and minor allele frequency (MAF) ≥0.05 in Caucasians and are located within 10 kb upstream of transcriptional start site and 10 kb downstream of transcriptional end site. For genes of less well-defined functional importance, only potentially functional SNPs from all two-hits in dbSNP database (http://www.ncbi.nlm.nih.gov/projects/SNP/) or HapMap database (http://www.hapmap.org) validated SNPs with an Illumina designability score ≥ 0.6 and MAF≥ 0.01 in Caucasians were included. A total of 587 SNPs in 65 genes of the apoptosis pathway were selected for genotyping.

Genomic DNA was isolated from peripheral blood using the QIAamp DNA Blood Maxi Kit (Qiagen, Valencia, CA) according to the manufacturer's protocol. A custom-designed panel of cancer-related genes had been generated in our lab, which covered 12 major cellular signaling pathways and 998 genes, including those in the apoptosis pathway [16]. Genotyping was carried out using Illumina's Infinium iSelect HD Custom Genotyping Beadchip according to the manufacturer's Infinium II assay protocol (Illumina, San Diego, CA) with 750 ng of input DNA for each sample. Genotyping data was then analyzed and exported using BeadStudio software (Illumina, San Diego, CA). The average call rate for the SNP array was >99.7%. Randomly selected 2% of samples were run in duplicates and the concordance of genotype calls was >99.9% for duplicate samples. All laboratory personnel were blinded to the case-control status of the study subjects.

### Statistical analysis

Statistical analyses were performed using the Intercooled Stata 10.0 statistical software package (StataCorp LP, College Station, TX). We performed Pearson chi-square test or Fisher's exact test to compare the difference in distribution of categorical variables such as genotype frequencies in cases and controls. For continuous variables, such as age, the Student's *t* test was used to test for differences between the case and control subjects. Unconditional multivariate logistic regression was applied to estimate the odds ratios (ORs) and 95% confidence intervals (95% CI) adjusted for age, where appropriate. Hardy-Weinberg equilibrium was tested for the genotypes using goodness-of-fit *X*^2^ test to compare the observed with the expected frequency of genotypes in controls. For each SNP, we tested its association with cancer risk in three different genetic models, dominant, additive and recessive models to define the best-fitting model with most significant P value. Only the result predicted by the best model was reported and considered in the subsequent analysis. If the percentage of the homozygous variant genotypes was less than five in cases or controls, we only considered the dominant model which has the highest statistical power. For internal validation, we generated a bootstrap resampling method for 100 times on samples randomly drawn from the original data set. Cumulative effects of multiple variants were analyzed by counting the number of unfavorable genotypes identified from the main effects analysis of single SNPs (P < 0.05). The unfavorable genotypes were divided into 4 groups (low-, medium-low, medium-high, and high-risk) according to the quartile of overall subject investigated. The reference group was that with the lowest risk. The high-order gene-gene interactions were explored via classification and regression tree (CART) analysis using Helix-Tree Genetics Analysis Software (Golden Helix, Bozeman, MT). CART uses recursive partitioning to build a decision tree that enables identification of subgroups of individuals at differential risks [43, 44]. We selected P-values to measure goodness of split and control tree growth (P <0.05). To control for multiple testing, q value (a false discovery rate (FDR)-adjusted P value) [45] was calculated for each SNP excluding those with strong linkage disequilibrium (r^2^>0.8) implemented in the R-package. We also performed 10,000 bootstrap runs to construct 95%CIs for the ORs in cumulative genotype analysis and CART analysis. All P values reported in this study were two sided.
